# Multi-pronged surveillance to understand the spatiotemporal correlations among macaques, vectors and humans in *Plasmodium knowlesi* malaria transmission

**DOI:** 10.1186/s13071-025-07082-6

**Published:** 2025-10-29

**Authors:** Wei Kit Phang, Nantha Kumar Jeyaprakasam, Sandthya Pramasivan, Zailiza Binti Suli, Jenn Zhueng Tam, Mohd Hafizi Bin Abdul Hamid, Mohd Lutfi Bin Abdullah, Anis Adlina Binti Isman Rohimly, Norsharina Binti Ashrat, Ting-Wu Chuang, Wang Nguitragool, Indra Vythilingam, Yee Ling Lau

**Affiliations:** 1 Department of Parasitology, Faculty of Medicine, Universiti Malaya, Kuala Lumpur, Malaysia; 2 Department of Molecular Tropical Medicine and Genetics, Faculty of Tropical Medicine, Mahidol University, Bangkok, Thailand; 3 Mahidol Vivax Research Unit, Faculty of Tropical Medicine, Mahidol University, Bangkok, Thailand; 4 Biomedical Science Program, Center for Toxicology and Health Risk Studies, Faculty of Health Sciences, Universiti Kebangsaan Malaysia, Kuala Lumpur, Malaysia; 5 Disease Control Division, Ministry of Health Malaysia, Putrajaya, Malaysia; 6 National Wildlife Forensic Laboratory, Ex-Situ Conservation Division, Department of Wildlife and National Parks Peninsular Malaysia, Kuala Lumpur, Malaysia; 7 Department of Molecular Parasitology and Tropical Diseases, School of Medicine, College of Medicine, Taipei Medical University, Taipei, Taiwan

**Keywords:** Epidemiology, Surveillance, *Plasmodium knowlesi*, One health, Zoonotic, Vectors, Non-human primates

## Abstract

**Background:**

*Plasmodium knowlesi*, a non-human primate (NHP) malaria parasite, has become a major public health concern in Malaysia and is now the leading cause of human malaria infections in the country. The transmission of *P. knowlesi* involves a complex cycle among humans, non-human primates and vectors. Numerous studies have focused on these hosts individually, but comprehensive research that integrates field data from all three hosts is lacking. This study aims to integrate multi-pronged surveillance data from macaques, vectors and human blood samples to better understand the epidemiology of *P. knowlesi* malaria in Peninsular Malaysia.

**Methods:**

Field sampling data (both previously published and unpublished) collected from humans, macaques and mosquito vectors by this research group in Peninsular Malaysia between 2019 and 2022 were integrated. The data collected for each host type within the same site and month were aggregated as a single sampling event. Partial correlations of outcomes between different host sampling sites were analysed by controlling for inter-host sampling site proximity and temporal difference. Spatiotemporal correlations were analysed between the sampling outcomes and historical human *P.* *knowlesi* malaria cases reported within defined distances (up to 20 km) from the sampling sites across different time lead windows (range from −12 to 12 months).

**Results:**

Partial correlation analysis, controlled for inter-host sampling-site spatial proximity and temporal difference, showed a statistically significant positive partial correlation between the proportion of field-sampled human *P. knowlesi*-positive cases and the average number of *Anopheles* Leucosphyrus-group mosquitoes sampled per night within a 10-km proximity constraint (*rs* = 0.228, *P* = 0.042). A consistently statistically significant positive correlation was found between the proportion of *P. knowlesi*-positive macaques and the number of historical human *P. knowlesi* cases reported in defined spatial proximity to macaque sampling sites, particularly within spatial radii of 6 km and beyond, across both backward and forward time leads. Other NHP malaria parasites, *P. cynomolgi*, *P. inui*, *P. coatneyi* and *P. fieldi*, exhibited heterogeneous patterns in macaques and vectors, particularly in terms of geographical distribution and mixed-species infection. The proportions of macaque samples positive for *P. knowlesi*, *P. inui* and *P. coatneyi* were statistically higher in the peridomestic–agriculture area as compared with the urban area.

**Conclusions:**

A key finding from this study is that the proportion of *P. knowlesi* infection in macaques may serve as a useful proxy for persistent transmission in an area, potentially indicating increased risk of human infection in nearby communities. This highlights the value of wildlife surveillance in predicting and managing zoonotic malaria risk. Integrating insights from epidemiology, ecology, veterinary science and public health is essential for effectively controlling zoonotic diseases such as *P. knowlesi* malaria and reducing their impact on both human and animal populations.

**Graphical abstract:**

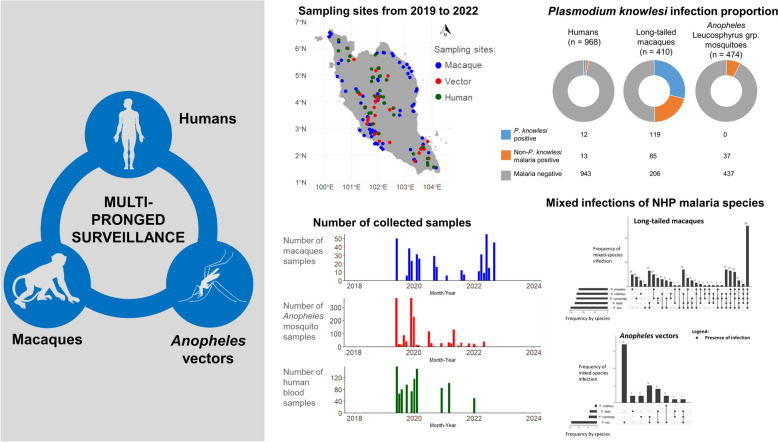

**Supplementary Information:**

The online version contains supplementary material available at 10.1186/s13071-025-07082-6.

## Background

In recent decades, malaria caused by *Plasmodium knowlesi* has emerged as a growing public health threat in Southeast Asia, with the number of cases steadily increasing each year. Although Malaysia reported the highest number of total *P. knowlesi* cases (2879 cases in 2023), there was also a significant increase in *P. knowlesi* cases in Thailand (from 71 cases in 2021 to 258 in 2022) and in Indonesia (from 5 cases in 2021 to 170 in 2023) [1]. *P. knowlesi* is a zoonotic malaria parasite that primarily infects non-human primates (NHPs), particularly macaques, and is transmitted to humans through the bite of infected *Anopheles* mosquitoes. The increasing overlap between human, NHP and vector habitats, coupled with changes in land use, has contributed to the increasing incidence of zoonotic malaria [2]. This rise in human *P. knowlesi* infections highlights the need to better understand the interactions among humans, macaques and mosquito vectors in Southeast Asia. The World Health Organization (WHO) reports that countries will not achieve malaria elimination status if the number of *P. knowlesi* cases remains high [3].

Several *Anopheles* mosquito species from the Leucosphyrus group, including *An. cracens*, *An. latens*, *An. balabacensis*, *An. hackeri* and *An. introlatus*, are incriminated vectors capable of transmitting *P. knowlesi* to humans [4–9]. These mosquito species have zoophilic tendencies and are predominantly exophagic and exophilic, differing from the typical vector of the human malaria parasite. These behaviours pose a challenge to current vector control strategies, which primarily target indoor-biting mosquitoes [10]. Hence, there is an urgent need for more effective vector control strategies. With growing reports and awareness of the potential public health burden of *P. knowlesi*, researchers have developed advanced methods to better understand the role of vectors in disease transmission. These include xenosurveillance to determine host preference from vector blood meals as well as geospatial mapping to estimate vector distribution [11–13]. Importantly, data generated from vector sampling provide valuable insights into the spatial and temporal dynamics of vector occurrence, vector bionomics, blood feeding preferences and ecological niche specificity.

Non-human primates (NHPs), such as long-tailed macaques, pig-tailed macaques and stump-tailed macaques, serve as natural reservoirs for *P. knowlesi* [14, 15]. Anthropogenic activities such as plantation development, farming, logging and deforestation in the past few decades may have altered the ecology and behaviour of *Anopheles* mosquitoes and NHPs [2]. For example, these changes can disrupt the natural habitats and food sources of macaques, forcing them to migrate to areas with a greater food supply, often near human settlements [16]. As a result, co-existence between macaques and humans becomes more frequent, leading to an increased risk of parasite transmission [17]. A previous seroprevalence study revealed a positive association between *P. knowlesi* exposure and contact with macaques [18].

To understand the transmission of zoonotic pathogens, it is crucial to integrate data on environmental changes, ecological relationships and pathogen circulation involving humans, reservoirs and vectors. Typically, studies on NHPs and vectors have been conducted separately at specific locations owing to the complexities involved in multi-sectoral research. This leads to limited analysis of the data on pathogen epidemiology circulating between humans, vectors and macaques. Integration of these data with geospatial analysis techniques into infectious disease epidemiology will improve surveillance reporting, spatiotemporal disease detection, identification of environmentally associated factors and prediction of pathogen and host distributions [13, 19–21]. For example, geospatial mapping has successfully identified larval habitats covering a large geographic area, which may be difficult or impossible to obtain through field surveys [22].

This study leveraged extensive field data encompassing mosquito sampling, macaque sampling and the documentation of human malaria cases to unravel the intricate web of *P. knowlesi* transmission. The primary goal was to investigate the interactions among humans, NHPs and mosquito vectors, shedding light on their roles in the transmission dynamics of zoonotic malaria. By taking a holistic approach, this study underscores the multifaceted nature of transmission, emphasising the influence of ecological, environmental and behavioural factors across multiple hosts.

## Methods

### Study locations

This study focused on Peninsular Malaysia, which spans from latitude 1° 15′ 50.0″ N to 6° 43′ 36″ N and from longitude 99° 35′ E to 104° 36′ E. Peninsular Malaysia covers a land area of 13.21 million hectares. Its administrative region is divided into 11 states (Perlis, Kedah, Pulau Pinang, Perak, Selangor, Negeri Sembilan, Melaka, Johor, Kelantan, Terengganu and Pahang) and two federal territories (Kuala Lumpur and Putrajaya).

### Screening for asymptomatic malaria in humans

Sampling was conducted in districts with previously reported malaria cases from 17 June 2019 through 22 January 2022. Study sites for participant recruitment were selected on the basis of previously reported malaria cases or the presence of high-risk groups, such as agricultural workers and army personnel who have high exposure to forested areas. Whole blood (5 mL) was collected from consenting participants in a lithium heparin blood-collection tube for screening via blood film examination and nested polymerase chain reaction (PCR). The initial round of nested PCR was initially conducted by targeting the 18S ribosomal ribonucleic acid (*18S rRNA*) of the *Plasmodium* genus. All samples positive for the *Plasmodium* genus were screened using nested PCR targeting the *18S rRNA* genes of *P. knowlesi*, *P. vivax*, *P. falciparum*, *P. ovale* and *P. malariae*. For samples with unidentified *Plasmodium* species, the genus amplicons of *18S rRNA* were amplified, cloned and sequenced [23, 24]. The generated sequences were validated through Basic Local Alignment Search Tool (BLAST) analysis against publicly available reference sequences in the National Center for Biotechnology Information (NCBI) database. This screening method has been described in detail [24, 25]. The protocol for human blood sampling was approved by the Medical Research and Ethics Committee (MREC), Ministry of Health Malaysia, and was registered with the National Medical Research Register, Ministry of Health Malaysia (approval no. NMRR-15–67223975).

### Historical human knowlesi malaria case data

The secondary data of confirmed *P. knowlesi* mono-infection cases between January 2018 and December 2023 were obtained from the Ministry of Health Malaysia database. According to the Ministry of Health, malaria cases are detected via active case detection (ACD), mass blood survey (MBS) and passive case detection (PCD). Most of the confirmed *P. knowlesi* cases in Peninsular Malaysia were determined through the PCD approach. All *P. knowlesi* malaria cases were confirmed by microscopy and PCR as described in the national malaria management guidelines [26]. The case data that were successfully geolocated and included onset date information were incorporated into the data analysis in this study.

### Macaque blood sampling

Macaque blood samples were collected as part of the Wildlife Disease Surveillance Programme (WDSP), by the Department of Wildlife and National Parks (DWNP). Macaques were trapped at sites where human–macaque conflict had been reported, typically in close proximity to human settlements. This sampling work was conducted from 17 June 2019 through 20 September 2022. All macaque blood withdrawal procedures were conducted under appropriate ethical approvals from the DWNP (ref no. W-00256-15−19) and the Universiti Malaya Institutional Animal Care and Use Committee (UM IACUC; ref no. M/06122019/25022019-01/R), in accordance with the Animal Research: Reporting of in Vivo Experiments (ARRIVE) guidelines [27]. DNA was extracted from 100 μL whole blood using the DNeasy^®^ Blood and Tissue kit (QIAGEN) according to the manufacturer’s protocol. Samples were initially screened using a *Plasmodium* genus nested PCR targeting the *18S rRNA* genes as described previously [28]. Samples positive for genus were screened using nested PCR targeting the *18S rRNA* genes of *P. knowlesi*, *P. cynomolgi*, *P. inui*, *P. coatneyi* and *P. fieldi* [29]. Detailed sampling methods and the corresponding data are available in a separate publication by the same research team [27]. Only long-tailed macaque samples were included in this study. Prior to analysis, all sampling sites were categorised into three landscape types: peridomestic–agriculture, peridomestic–forest and urban areas.

### *Anopheles* mosquito sampling data

The mosquitoes were collected from 17 June 2019 to 1 May 2022. Mosquitoes were collected via human landing catch (HLC) and Mosquito Magnet. The protocol for HLC was approved by the Medical Research and Ethics Committee, Ministry of Health Malaysia (approval no. NMRR-19–962−47,606). All the collected *Anopheles* mosquitoes were morphologically identified to the species level using the taxonomic keys of Reid [30] and Sallum et al. [31]. *Anopheles* mosquitoes from the Leucosphyrus group, as well as those that were difficult to identify morphologically, were confirmed molecularly through DNA sequencing of the *ITS2* gene [12, 32]. Detailed methods and the corresponding raw data are available in separate publications by the same research team [12, 32].

### Human knowlesi malaria risk and *Anopheles* Leucosphyrus-group mosquito distribution map data

Several datasets were generated within the same study period by our research team. This includes the generation and publication of a predicted human knowlesi malaria risk map [21] and a predicted distribution map of *Anopheles* Leucosphyrus-group mosquitoes [12]. The human knowlesi malaria risk map was generated using Extreme Gradient Boosting (XGBOOST) by training on geolocated human knowlesi malaria cases from 2011 to 2018. The predicted map of distribution of *Anopheles* Leucosphyrus-group mosquitoes was generated using logistic regression of primary and published data assembled from 1957 to 2021. The application of disease risk and mosquito distribution maps was compared and assessed against the progression of new cases reported from 2020 to 2022. In addition, this map was also used to assess the distribution of sampling sites and their outcomes in this study.

### Data analysis

We consider a ‘sampling event’ as the collection of samples within a single month at the same site. Therefore, the data collected for each host type within the same site and the same month of the year were aggregated as a single sampling event. The use of multiple methods in sampling mosquitoes, which include HLC and Mosquito Magnet, increases the difficulty of calculating human biting rate (i.e. the number mosquitoes sampled per person per night); hence, we calculated the average number of mosquitoes sampled per night in each month for all *Anopheles* mosquitoes and *Anopheles* Leucosphyrus-group mosquitoes.

All data analyses were conducted using R, version 4.2.3, and QGIS, version 3.34.9. Euclidean distances between different host sampling sites (human, macaque and mosquito) were calculated to determine their spatial proximity. Host sampling site pairs within a maximum distance of 40 km were identified (Additional File 1; Supplementary Fig. S1). Partial correlation analyses were then performed for host pairs within three proximity constraints: 10 km, 20 km and 40 km. For each pair, the temporal difference in months was also calculated. These analyses were conducted as part of a sensitivity assessment across different proximity constraints. Partial correlation analysis using Spearman’s correlation method was conducted to identify pairwise correlation between sampling outcomes of different host components. In this analysis, both spatial proximity and temporal difference between host sampling sites were controlled for.

For each sampling event, the number of historical human *P. knowlesi* infection cases reported within specific distances from the site  was counted at varying radii with 1-km interval up to a maximum of 20 km across different time leads measured in months (Additional File 1; Supplementary Text S1 and Supplementary Fig. S2). Spatiotemporal correlations between the sampling outcomes and the historical human *P. knowlesi* infection cases were analysed using Spearman’s correlation test. To account for the increased likelihood of type I errors due to conducting multiple correlation tests, we applied a false discovery rate (FDR) adjustment to the *P* values. This method ensures that the results are more robust and reduces the chances of identifying spurious correlations due to multiple statistically significant tests.

In addition, we calculated both backward and forward cumulative numbers of historical *P. knowlesi* infections in relation to the sampling dates (Additional File 1: Supplementary Text S1 and Supplementary Fig. S3. Spatiotemporal correlation tests were then performed between the sampling outcomes and, separately, the backward and forward cumulative number of human knowlesi malaria cases. The rationale for examining spatiotemporal correlations with human infection records prior to the sampling event, including both independent temporally lagged data (negative time leads) and backward cumulative number of human knowlesi malaria cases, is to investigate whether past human infection trends are associated with current infection prevalence in asymptomatic humans, macaques or mosquitoes, potentially indicating persistent local transmission. Conversely, analysing spatiotemporal correlations with human infection records following the sampling event, including both independent temporally led data (positive time leads) and forward cumulative number of human knowlesi malaria cases, aims to assess whether infections detected in macaques or mosquitoes can serve as predictors of future human cases, thereby offering early warning potential.

The outcomes of the sampling were analysed and compared across the predicted maps generated in this research group. An UpSet plot was plotted to illustrate the pattern and frequency of mixed infection of NHP malaria species. Pearson’s *χ*^2^ tests were used to compare the proportions between groups, followed by multiple pairwise comparison tests with Bonferroni’s correction. For all statistical tests, a *P* value ≤ 0.05 was identified as statistically significant. The R code for the data analysis in this study is available at https://github.com/WKPhang/pk-multi-pronged-peninsular-malaysia-lrgs.

## Results

### Outcomes of multi-pronged surveillance

In the human blood sampling study, 968 participants provided consent and had their blood sampled across 42 sampling events. A total of 25 samples tested positive for asymptomatic *Plasmodium* infections, including *P. knowlesi* (*n* = 12; 1.2%), *P. inui* (*n* = 3; 0.3%), *P. vivax* (*n* = 8; 0.8%), *P. ovale* (*n* = 1; 0.1%) and unspecified *Plasmodium* (*n* = 1; 0.1%). All samples were ruled as sub-microscopic except for two *P. inui* samples, which were found to be likely *Plasmodium* trophozoites during 2 h of microscopic examination [24]. Notably, data from 17 June 2019 to 8 January 2020 (*n* = 585) were documented in a previous publication [25], and the remaining 383 samples were newly generated in this study.

**Fig. 1:**
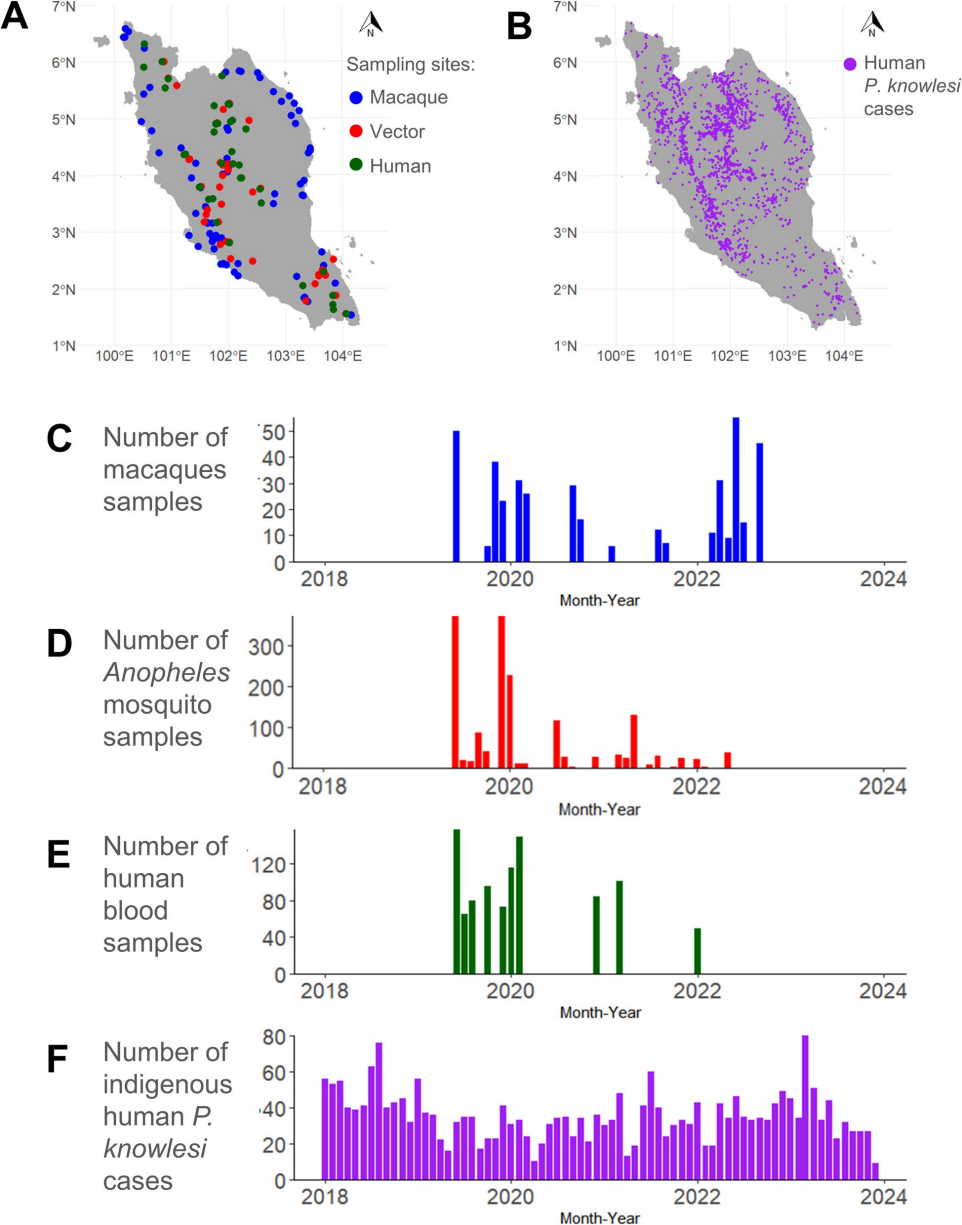
Geographical distribution of sampling sites (**A**) and indigenous human *P. knowlesi* cases from 2018 to 2023 (**B**). Time-series of distribution of the number of samples collected by type of host (**C–E**) and the number of reported indigenous human *P. knowlesi* cases (**F**) throughout the study period

In macaque blood sampling, 410 long-tailed macaques were sampled across 78 sampling events. A total of 204 long-tailed macaques were positive for the malaria parasite. These macaques were positive for *P. knowlesi* (*n* = 119; 29.0%), *P. cynomolgi* (*n* = 128; 31.2%), *P. inui* (*n* = 152; 37.1%), *P. coatneyi* (*n* = 127; 31.0%) and *P. fieldi* (*n* = 135; 32.9%), which is similarly described in a previous publication [27]. Meanwhile, for vector sampling, 1652 *Anopheles* mosquitoes were collected across 69 sampling events, of which 474 (28.7%) belonged to the Leucosphyrus group. Among these 474 *Anopheles* Leucosphyrus-group mosquitoes, 37 (7.8%) were found to be positive for *Plasmodium* species by midgut or salivary gland PCR screening. These mosquitoes were positive for *P. cynomolgi* (*n* = 9; 1.9%), *P. inui* (*n* = 29; 6.1%), *P. coatneyi* (*n* = 2; 0.4%) and *P. fieldi* (*n* = 8; 1.7%). Three of the *Anopheles* Leucosphyrus-group mosquitoes were found to be positive for *Plasmodium* sp., but their species could not be identified. In addition, three *An. letifer* of Umbrosus-group mosquitoes were found to be positive for *Plasmodium* sp. with unidentified species. All percentages reported for *Plasmodium* infection in mosquitoes are based on the total number of *Anopheles* Leucosphyrus-group mosquitoes collected. The comparison of the spatial and temporal distribution of sampling events for these three hosts with the reported human *P. knowlesi* cases across Peninsular Malaysia is shown in Fig. 1.

### Partial correlation between sampling outcomes

The human sampling sites and vector sampling sites were in closer proximity as compared with macaque sampling sites (Additional File 1: Supplementary Table S1). On the basis of the selected proximity constraints of 10, 20 and 40 km, there was no statistically significant correlation between human and macaque sampling outcomes after controlling for proximity and temporal difference. Moderate statistically significant positive correlations were observed between the proportion of malaria-positive cases in humans and the sampling outcomes of vectors. Within 10-km proximity constraints, the proportion of malaria-positive cases in humans was positively associated with the average number of *Anopheles* mosquitoes sampled per night (*rs* = 0.433, *P* < 0.001), the average number of *Anopheles* Leucosphyrus-group mosquitoes sampled per night (*rs* = 0.590, *P* < 0.001) and the proportion of malaria-positive cases in *Anopheles* mosquitoes (*rs* = 0.476, *P* < 0.001). These correlations remained significant, although with decreasing effect, at 20-km and 40-km proximity constraints. In addition, statistically significant positive partial correlations between the proportion of human asymptomatic *P. knowlesi*-positive cases and the average number of *Anopheles* Leucosphyrus-group mosquitoes sampled per night were observed within a 10-km proximity constraint (*rs* = 0.228, *P* = 0.042). A similar trend was found for the malaria-positive proportion in macaques and average number of *Anopheles* mosquitoes sampled per night (*rs* = −0.493, *P* = 0.020 within 10 km). Positive correlations between the macaque *P. knowlesi*-positive proportion in macaques and the average number of *Anopheles* Leucosphyrus-group mosquitoes sampled per night emerged at larger distances (*rs* = 0.194, *P* = 0.003 at 40 km).

### Spatiotemporal correlation between sampling outcomes and reported human knowlesi cases

**Fig. 2:**
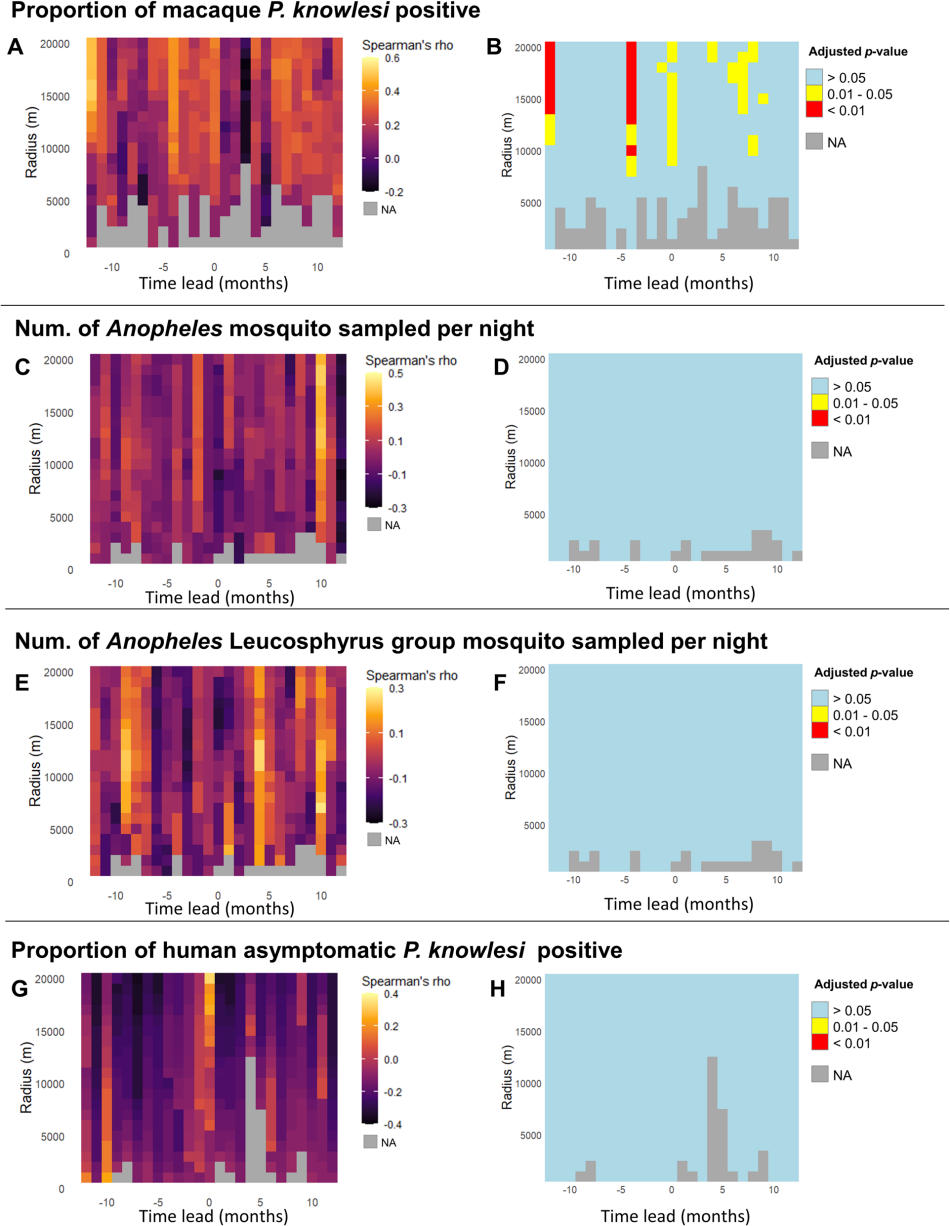
Spatiotemporal correlation between reported human *P. knowlesi* cases (within a specific radius and different time lead from sampling event) and respective sampling outcomes. These sampling outcomes include the proportion of macaque *P. knowlesi*-positive cases (**A**) and its associated statistical significance plot (**B**), number of *Anopheles* mosquitoes sampled per night (**C**) and its associated statistical significance plot (**D**), number of *Anopheles* Leucosphyrus-group mosquitoes sampled per night (**E**) and its associated statistical significance plot (**F**) and proportion of human asymptomatic *P. knowlesi*-positive cases (**G**) and its associated statistical significance plot (**H**). The *P* values were adjusted using the FDR approach. Negative time leads are equivalent to time lags, i.e. the number of months prior to the sampling event month. NA indicates that correlation coefficient was not computed owing to the absence of cases counted within the radius at the specified time

There were statistically significant positive correlations between the historical number of human *P. knowlesi* malaria cases and the proportion of *P. knowlesi*-positive macaques, particularly at a time lead of −12 months within at least 11 km (*rs* = 0.331–0.517), −4 months within at least 8 km (*rs* = 0.329–0.406), 0 months (sampling month) within 9 km (*rs* = 0.316–0.374) and between 6 and 8 months within at least 9 km (*rs* = 0.310–0.363) (Fig. 2A and B). However, there was no statistically significant correlation between the number of mosquitoes sampled per night and human knowlesi malaria case number within any radius up to 20 km at any time (Fig. 2C–H).

Spatiotemporal correlation analysis involving backward cumulative historical human *P. knowlesi* infection cases revealed statistically significant correlations at broader spatial radii, particularly from 6 km and beyond, across all tested time leads (Additional File 1: Supplementary Fig S4A and C). This suggests that a higher proportion of *P. knowlesi*-positive macaques tends to correlate with previous increases in human cases, possibly reflecting potential persistent local transmission. Similarly, in the spatiotemporal correlation analysis between the proportion of *P. knowlesi*-positive macaques and forward cumulative human case numbers, consistent positive correlations were observed at radii of 6 km and beyond (Additional File 1: Supplementary Fig S4B and D). This indicates that a high proportion of *P. knowlesi*-positive macaques may also precede increases in reported human cases, suggesting their potential predictive value or role as an early reservoir.

### Comparison of the predicted risk map of knowlesi malaria and the distribution of *Anopheles* Leucosphyrus-group mosquitoes

**Fig. 3:**
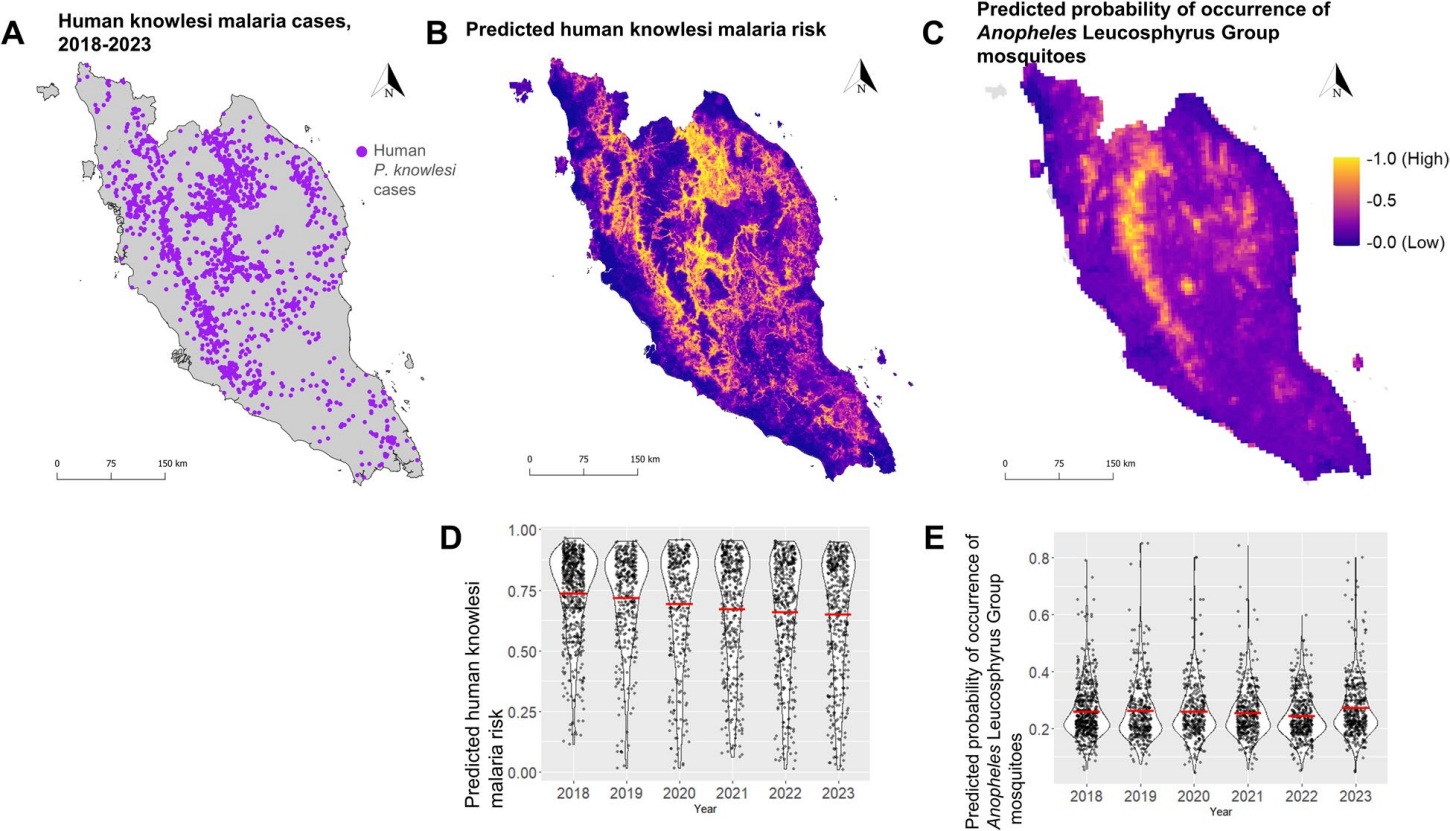
Map of reported knowlesi malaria cases (**A**) in comparison to previously predicted maps of human knowlesi malaria risk (**B**) and distribution of *Anopheles* Leucosphyrus-group mosquitoes (**C**). Violin plots indicating the overlapping of geolocated knowlesi malaria cases (year 2018–2023) with the predicted human knowlesi malaria risk map (**D**) and the predicted probability of occurrence of *Anopheles* Leucosphyrus Group-mosquitoes map (**E**). The *y*-axis on (**D** and **E**) indicate the predicted probability of human knowlesi malaria risk and occurrence of mosquitoes, respectively

The predicted human knowlesi malaria risk map (Fig. 3B) generated by XGBOOST model [21] has shown relatively stable prediction of new human knowlesi malaria cases in 2020–2023, as cases in 2018 and 2019 were involved in the model training and testing (Fig. 3D) (Table 1). On the contrary, human knowlesi malaria cases from 2018 to 2023 were predominantly distributed in the areas with a low probability of *Anopheles* Leucosphyrus-group occurrence. Comparing both these risk maps, the vectors are estimated to be abundant in deep inland, particularly in the mountainous area of Titiwangsa range (Fig. 3C), whereas the risk of human knowlesi malaria appears to be at lower elevation close to the mountain range (Fig. 3B). Both generated maps showed moderate positive correlation with the proportion of *P. knowlesi* infection in long-tailed macaques (Table 2). However, high-risk areas do not correlate with vector presence and detection of asymptomatic human infections at sampling sites (Table 2).

**Table 1:** Partial correlation analysis of sampling outcomes between any two host sampling outcomes controlled for temporal difference in month and proximity between sampling sites. The number of site pairs was calculated on the basis of Euclidean distances among human, macaque and mosquito sampling sites within each proximity constraint (Additional File 1: Supplementary Fig. S1)

Proximity constraint		Within 10 km		Within 20 km		Within 40 km	
First host variables	Second host variables	*rs*^a^	*P* value	*rs*^a^	*P* value	*rs*^a^	***P*** value
Humans	Macaques						
Number of site pairs		9		34		130	
*P. knowlesi*-positive proportion	*P. knowlesi*-positive proportion	−0.033	0.945	−0.151	0.408	−0.109	0.219
*P. knowlesi*-positive proportion	Number of macaques	0.164	0.725	0.159	0.383	0.063	0.478
Humans	Vectors						
Number of site pairs		82		119		239	
Malaria-positive proportion	Average number of *Anopheles* mosquitoes sampled per night	0.433	< 0.001^*^	0.287	0.002^*^	0.279	< 0.001^*^
Malaria-positive proportion	Average number of *Anopheles* Leucosphyrus-group mosquitoes sampled per night	0.590	< 0.001^*^	0.414	< 0.001^*^	0.268	< 0.001^*^
Malaria-positive proportion	Malaria-positive proportion	0.476	< 0.001^*^	0.342^*^	< 0.001^*^	0.237	< 0.001^*^
*P. knowlesi*-positive proportion	Average number of *Anopheles* mosquitoes sampled per night	0.203	0.071	0.146	0.116	0.067	0.316
*P. knowlesi*-positive proportion	Average number of *Anopheles* Leucosphyrus group mosquitoes sampled per night	0.228	0.042^*^	0.177	0.056	0.090	0.169
*P. knowlesi*-positive proportion	Malaria-positive proportion	0.193	0.086	0.151	0.105	0.070	0.282
*P. knowlesi* case number	Average number of *Anopheles* mosquitoes sampled per night	0.192	0.088	0.133	0.151	0.060	0.359
*P. knowlesi* case number	Average number of *Anopheles* Leucosphyrus-group mosquitoes sampled per night	0.211	0.060	0.159	0.086	0.078	0.232
Macaques	Vectors						
Number of site pairs		24		77		228	
Malaria-positive proportion	Average number of *Anopheles* mosquitoes sampled per night	−0.493	0.020^*^	−0.228	0.049^*^	−0.050	0.452
Malaria-positive proportion	Average number of *Anopheles* Leucosphyrus-group mosquitoes sampled per night	−0.064	0.778	0.045	0.700	0.188	0.005^*^
Malaria-positive proportion	Malaria-positive proportion	−0.302	0.172	−0.091	0.437	0.041	0.544
*P. knowlesi*-positive proportion	Average number of *Anopheles* mosquitoes sampled per night	−0.435	0.043^*^	−0.196	0.093	−0.097	0.147
*P. knowlesi*-positive proportion	Average number of *Anopheles* Leucosphyrus-group mosquitoes sampled per night	0.003	0.991	0.018	0.878	0.194	0.003^*^
*P. knowlesi*-positive proportion	Malaria-positive proportion	−0.284	0.200	−0.163	0.163	0.058	0.388

^a^Partial Spearman’s correlation coefficient

**Table 2:** Correlation analysis between surveillance outcomes and predicted maps in this research. Correlation plots for each analysis are available in Additional File 1: Supplementary Fig. S5

Host	Variables	Predicted human knowlesi malaria risk map [35]		Predicted probability of occurrence of *Anopheles* Leucosphyrus-group mosquitoes [12]	
		*rs*^a^	*P* value	*rs*^a^	*P* value
Macaque	Number of malaria positive cases	0.517	< 0.001^*^	0.409	< 0.001^*^
	Proportion of malaria-positive cases	0.485	< 0.001^*^	0.453	< 0.001^*^
	Number of *P. knowlesi*-positive cases	0.581	< 0.001^*^	0.405	< 0.001^*^
	Proportion of *P. knowlesi*-positive cases	0.611	< 0.001^*^	0.380	< 0.001^*^
Vector	Average number of *Anopheles* mosquitoes sampled per night	−0.263	0.030^*^	−0.140	0.251
	Average number of *Anopheles* Leucosphyrus-group mosquitoes sampled per night	−0.165	0.178	−0.149	0.222
	Number of malaria-positive cases	−0.312	0.010^*^	−0.117	0.362
	Proportion of malaria-positive cases (out of all *Anopheles* mosquitoes)	−0.286	0.018^*^	−0.068	0.576
Human	Number of asymptomatic *P. knowlesi*-positive cases	−0.191	0.226	−0.270	0.083
	Proportion of asymptomatic *P. knowlesi*-positive cases	−0.205	0.192	−0.264	0.091

^a^Spearman’s correlation coefficient

### Non-knowlesi NHP malaria surveillance

**Fig. 4:**
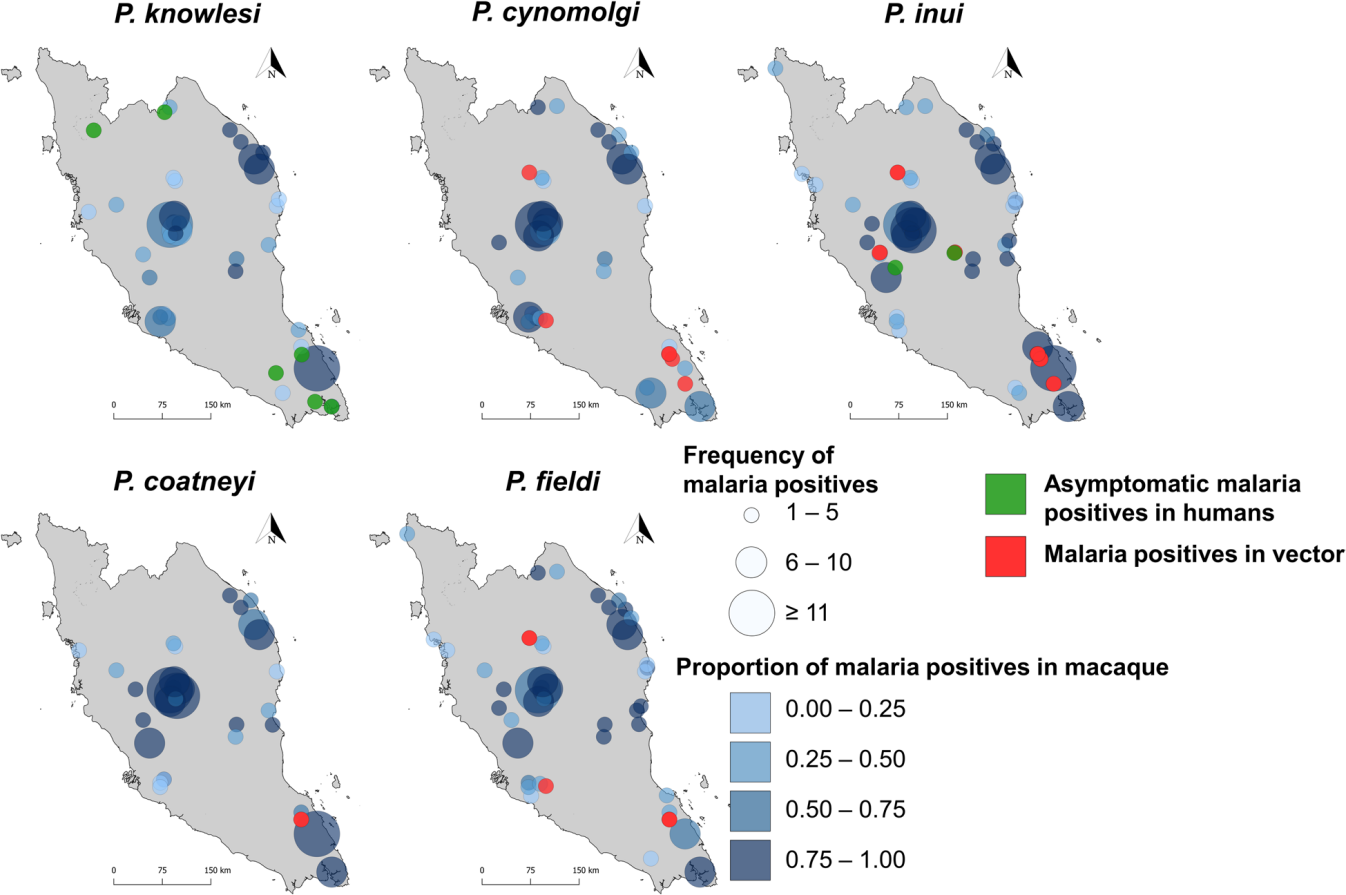
Distribution of sampling sites with knowlesi and other non-knowlesi malaria-positive samples

Among human blood screenings, three cases of *P. inui* were detected, with two of them previously reported in Maran, Pahang [24]. The majority of asymptomatic *P. knowlesi* infections in humans were found near the northern border and the southern region (Johor state) of Peninsular Malaysia (Fig. 4). Although there was a higher concentration of NHP malaria infections in long-tailed macaques in the central region, the number of positive infections in vectors in this area was low (Fig. 4). In addition, we reported *P. cynomolgi* and *P. inui* infections in mosquitoes. There was a high concentration of five NHP malaria species infection in macaques on the east coast; however, no human blood and vector sampling were conducted in this area.

**Fig. 5:**
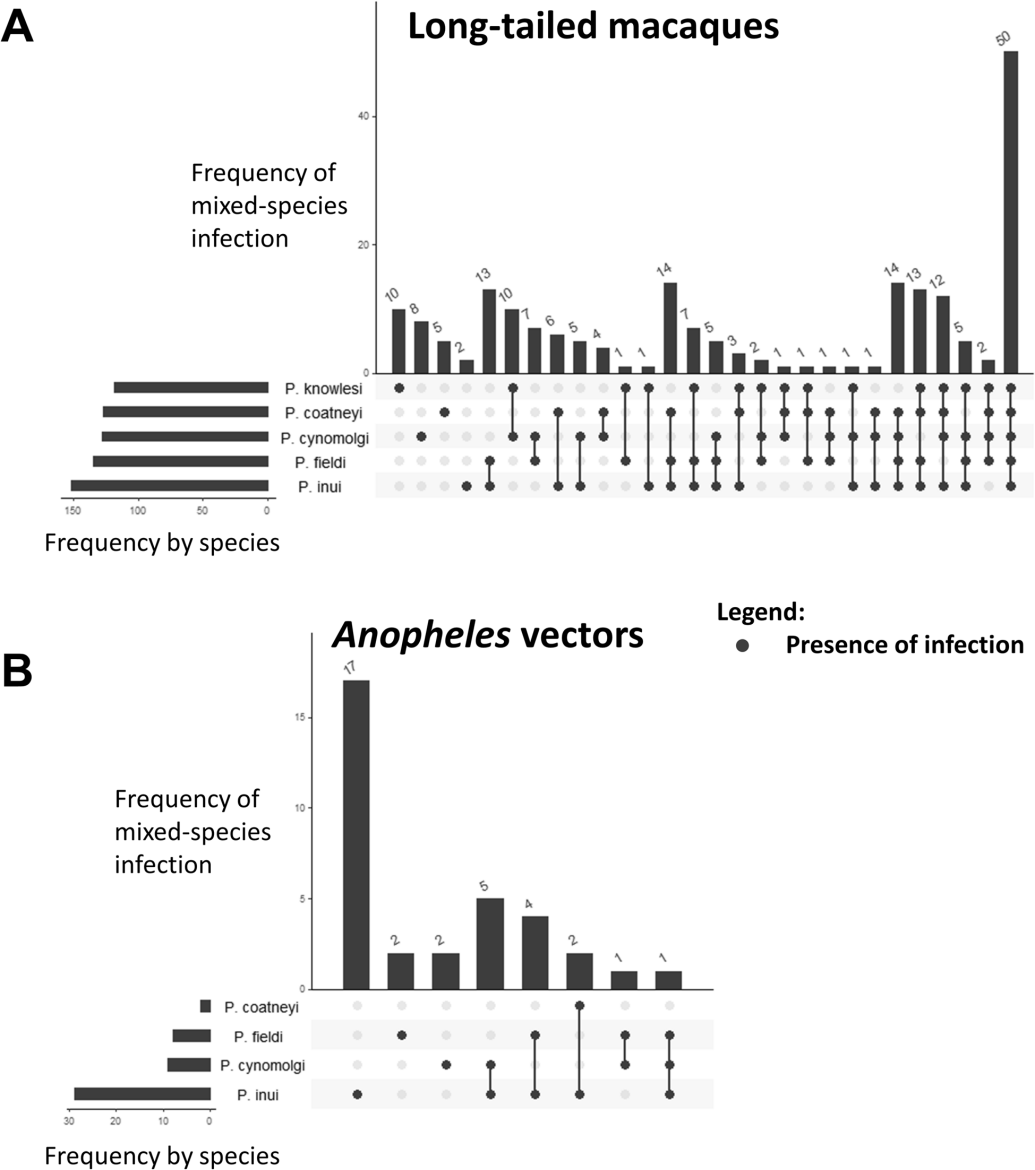
UpSet diagram illustrates the frequency of mixed-species infections in infected long-tailed macaques (*n* = 206) (**A**) and *Anopheles* vectors (*n* = 34) (**B**). The bar graphs to the left indicate the number of infections detected by *Plasmodium* species. The connected nodes indicate multiple infections by different *Plasmodium* species

Differential pattern of mixed-species infection was observed between long-tailed macaques and *Anopheles* mosquitoes (Fig. 5). The predominant type of infection in macaques out of all infected individuals was quintuple infection of NHP *Plasmodium* (*n* = 50; 24.3%) (Fig. 5A). Contrastingly, the major type of infection among infected *Anopheles* vectors was *P. inui* mono-infection (*n* = 17; 50.0%) (Fig. 5B).

### Comparing NHP *Plasmodium* species prevalence in long-tailed macaques across different landscapes

**Fig. 6:**
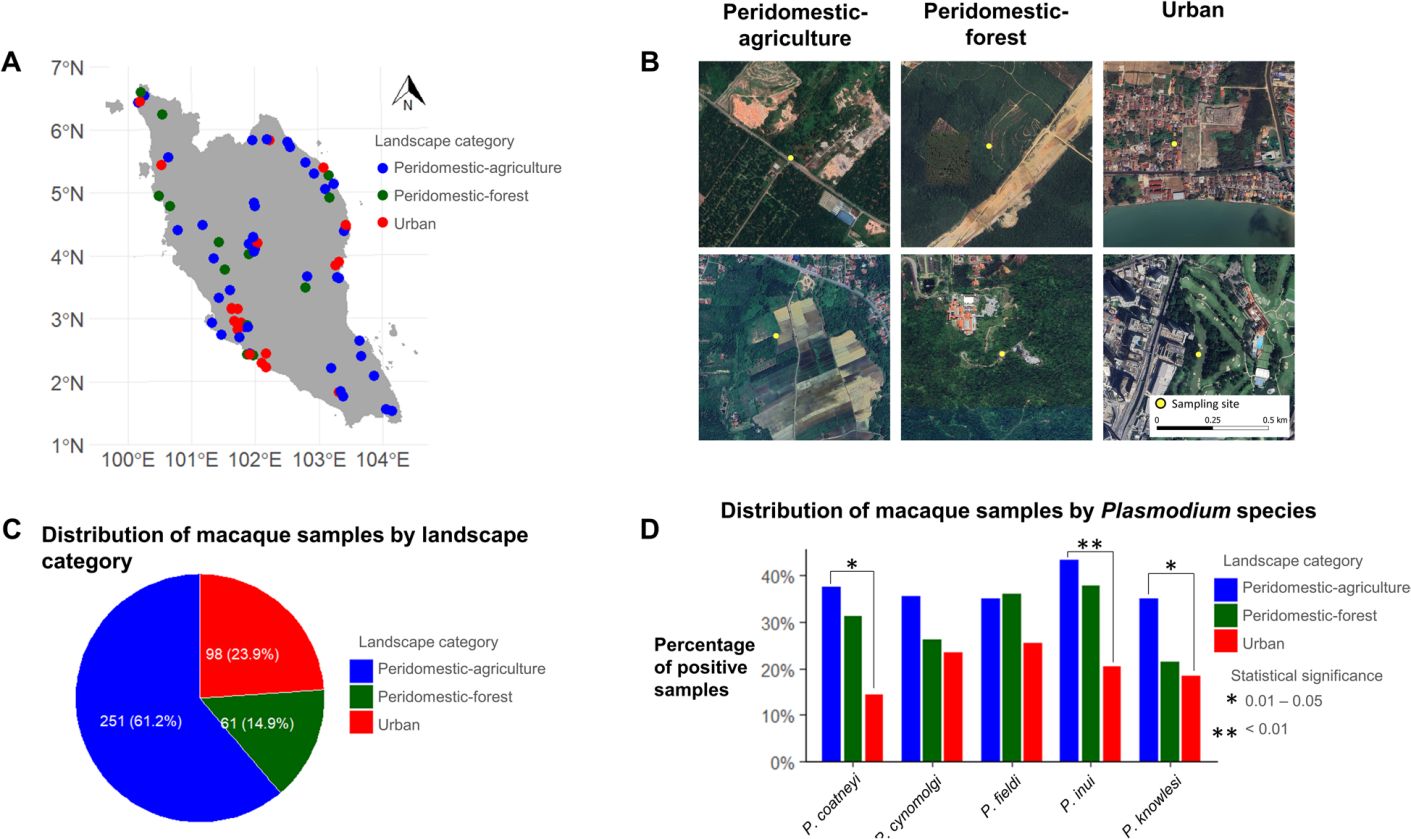
Geographical distribution of long-tailed macaque samples by landscape category (**A**). Example of what landscape category looks like based on satellite imagery (**B**). The base map was a Google Satellite image inputted and viewed on QGIS, version 3.34.9. The number of collected long-tailed macaque samples by landscape category (**C**). The proportion (in percentage) of infected samples by *Plasmodium* parasite species and landscape category (**D**)

Most long-tailed macaques were sampled in the peridomestic–agriculture area (Fig. 6). Besides, the proportion of the long-tailed macaques detected positive is generally higher in peridomestic areas as compared with urban areas (Fig. 6D). Significant differences were observed in the proportion of *P. knowlesi* (*χ*^2^ = 11.60, *df* = 2, *P* = 0.003), *P. inui* (*χ*^2^ = 16.02, *df* = 2, *P* < 0.001) and *P. coatneyi* (*χ*^2^ = 17.69, *df* = 2, *P* < 0.001) but not for *P. cynomolgi* (*χ*^2^ = 5.55, *df* = 2, *P* = 0.06) and *P. fieldi* (*χ*^2^ = 3.23, *df* = 2, *P* = 0.20) (Fig. 6D). Multiple pairwise comparison test showed that long-tailed macaque samples in peridomestic–agriculture landscape have a significantly higher proportion of *P. knowlesi* (adjusted (adj.) *P* = 0.011), *P. inui* (adj. *P* < 0.001) and *P. coatneyi* (adj. *P* < 0.001) than those in urban landscape (Fig. 6D).

## Discussion

We identified significant positive correlations between the proportion of *P. knowlesi* infections in macaques and the number of human knowlesi malaria cases recorded within a 20-km radius of the macaque sampling sites during the same month. This finding suggests a strong spatial and temporal link between non-human primate reservoirs and human cases of zoonotic malaria. The close proximity of NHPs, particularly long-tailed macaques in this case, to human populations could increase the risk of zoonotic transmission. The fact that these correlations were observed within the same month indicates that the transmission cycle of *P. knowlesi* is very localised by time, with human cases emerging alongside detection of the parasite in local macaque populations. The spatiotemporal correlations involving backward and forward cumulative of *P. knowlesi* infections in humans are most evident at least within a 6-km radius from macaque sampling sites, suggesting that macaque sampling outcomes could reflect both retrospective and prospective indications of *P. knowlesi* transmission in humans. This supports the hypothesis that macaques may act as reservoirs or indicators of environmental risk conditions relevant to human infection dynamics, with implications for predictive surveillance and spatial targeting of interventions. These findings highlight the importance of monitoring *P. knowlesi* infection rates in both macaque and human populations as part of a broader strategy to predict and manage outbreaks of zoonotic malaria. These data underscore the need for targeted interventions in areas where macaques and humans coexist, particularly within a short temporal frame from when positive infections are found.

The high proportion of *P. knowlesi* infections in macaques observed in areas identified as high risk for human *P. knowlesi* malaria is consistent with expectations, given the elevated incidence of human cases in these areas. This correlation could be due to areas with a high prevalence of infected macaques providing a larger reservoir for the parasite. Thus, the risk of transmission to humans via mosquito vectors increases. Understanding this relationship is crucial because it underscores the importance of targeting both macaque populations and mosquito vectors in high-risk areas to reduce the overall transmission of *P. knowlesi* to humans. It also emphasises the need for localised intervention strategies that consider the ecological and environmental factors contributing to the high prevalence of zoonotic malaria in these regions. Findings from this study demonstrate how integrative data analytics can support and enhance the optimization of current zoonotic malaria control strategies. This includes prioritizing surveillance and health education in areas where *P. knowlesi*-infected macaques and vectors co-occur near human settlements, as well as guiding targeted vector control efforts.

Although a high number of *Plasmodium* co-infections were observed in macaques, mosquitoes relatively more often harboured mono-infections of individual *Plasmodium* species; for example, *P. inui* mono-infections accounted for 17 cases (50%). These differences suggest that co-infections in macaques do not necessarily translate into co-infections in mosquitoes. One possible explanation is interspecific competition among *Plasmodium* species during gametocyte development within the mosquito midgut, which can result in the selective suppression of one species over another. This phenomenon has been demonstrated in avian *Plasmodium* species [33]. Nevertheless, co-infections in mosquitoes for zoonotic *Plasmodium* are not uncommon and have been reported in numerous previous studies [34]. In addition, differences in vector competence among *Anopheles* species may also influence infection patterns. Vector competence refers to the mosquito’s ability to acquire a parasite, support its development and ultimately transmit it to a new host. These species-specific differences can affect the likelihood of successful co-infection, as some *Anopheles* species may only be permissive to certain *Plasmodium* species.

The low detection of asymptomatic *P. knowlesi* infections in humans, despite the presence of competent *Anopheles* mosquitoes and a high prevalence of *P. knowlesi* in macaques near high-risk areas, should be interpreted with caution. This is because current surveillance systems may fail to detect low-density infections, especially in asymptomatic individuals who are less likely to seek testing. Therefore, the seemingly low number of asymptomatic cases does not exclude the possibility of ongoing silent transmission. The presence of past symptomatic cases in these areas further supports evidence of active transmission. One way to improve future mass blood screening efforts is to identify sites where infections are regularly reported among inhabitants. Another approach is to determine peak transmission periods when the highest number of cases occurs so that sampling efforts can be heightened or prioritised during this period. A separate study revealed that transmission was typically greater from January to March on the basis of 2012–2018 data from the central-northern region of Peninsular Malaysia [35]. A seasonal pattern of *P. knowlesi* malaria has also been observed in Sarawak, where case reports were consistently greater in October [19].

External pressures such as vector interventions, deforestation and landscape changes drive shifts in *Anopheles* vector composition, abundance, biting behaviour and physiology. Globally, malaria vectors have demonstrated the ability to adapt their biting location and biting time in response to selection pressures from vector control interventions [36, 37]. Landscape changes have also influenced the abundance of certain *Anopheles* species, and this has been particularly reported in East Malaysia. For instance, a study in Kinabatangan, Sabah, found that *An. donaldi* substituted *An. balabacensis* as the *P. falciparum* primary vector following deforestation and implementation of malaria vector control measures [38]. Meanwhile, a separate study in Miri, Sarawak, reported a 92% decline in *An. donaldi* abundance over 4 years, as forests were converted into oil palm plantations and settlements [39]. In a separate publication resulting from this research initiative, we reported that the number and species composition of *Anopheles* mosquitoes varied substantially across different sampling times over a 2-year period at similar locations [32]. For instance, the man-biting rate of *An. introlatus* sampled in Bukit Tinggi in September 2019 was at least 11 times lower than in the adjacent sampling events conducted in June 2019 and January 2020 [32]. Given this natural variability, it remains uncertain whether vector composition will continue to change in the future, particularly for NHP malaria vectors. Therefore, accounting for temporal effects in our current analysis is a necessity, as vector abundance and species composition can vary considerably over short timeframes, potentially influencing transmission patterns.

However, unlike human blood survey and macaque sampling, entomological surveillance presents several unique challenges, as this work usually involves the deployment of HLC that directly exposes field sampling staff to vector bites [40–42]. Vector sampling often involves reactive mosquito surveillance, where sampling efforts are intensified in response to reported malaria case clusters. This approach, while essential for identifying transmission hotspots, can be logistically demanding, requiring rapid mobilization of field teams, coordination with local health authorities, and adaptation to varying environmental conditions. Therefore, entomological studies and routine surveillance should consider other alternatives such as Mosquito Magnets, as they have been proven to be effective [41]. In contrast, it is essential to recognise that vector control interventions in public health settings are typically implemented following entomological surveillance [43]. This sequence of activities can introduce variability in mosquito abundance and species composition, particularly in the same localities where research sampling is conducted after interventions have already been implemented. This may help explain the lack of observed spatiotemporal correlation between *Anopheles* mosquito abundance and historical human *P. knowlesi* case counts in our study.

As a country’s malaria disease burden decreases to very low levels along with advancements in molecular surveillance and diagnostics, asymptomatic NHP malaria cases are detected in humans. It is crucial to reconsider the threat of NHP malaria and establish monitoring and control strategies. Surveying both macaques and mosquitoes plays an important role in zoonotic vector-borne diseases, as this could decipher complex relationships. Studies in our research group have shown that the predominant species found in long-tailed macaques and vectors are *P. inui* and *P. cynomolgi* [27, 32]. However, only sporadic human infections with these parasites have been reported, and most were initially detected through sequencing following genus-specific PCR, rather than by species-specific PCR. This discrepancy underscores a potential under-detection in humans and supports the need for broader species screening in routine surveillance.

This study highlights the need for future human screening to include all five NHP malaria species. Hence, surveillance and mapping the distribution of NHP malaria vectors is essential for the implementation of effective surveillance and control measures to eliminate the disease. Comprehending the spatial and temporal patterns of NHP malaria prevalence in both macaque and vectors is crucial, as it helps identify hot-spot areas of sustained disease transmission, and resources can be allocated effectively.

This study has limitations. First, the distance between some of the sampling sites may have been too great to accurately reflect the geographical associations between the datasets. The selection criteria for these sampling sites differed: NHP sampling was based on areas where human–macaque conflicts occurred, while human blood and vector sampling focused on locations near previously reported human knowlesi malaria cases. This discrepancy led to variability in sampling periods across different locations, making it challenging to draw definitive conclusions about the association between entomological indices and the specific sites where macaques were captured and screened. In addition, reported household case locations may not necessarily represent the actual sites of infection. In many instances, individuals acquire infections while working or venturing into forested or deep jungle areas to collect forest products. These areas are often inaccessible for mosquito sampling using HLC owing to logistical constraints and the inherent risks associated with wildlife encounters. As a result, the study’s ability to directly correlate vector presence with the proportion of NHP infection in the same locations was limited.

Given the growing importance of understanding sylvatic cycles of zoonotic malaria in an environment of rapid anthropogenic disturbance, various surveillance methods, particularly more localised surveillance methods, require further consideration. Monitoring macaque movement, including cross-border migration, remains a knowledge gap that could contribute to the understanding of disease transmission dynamics. It has been hypothesised that macaques may carry malaria parasites across borders, potentially contributing to rising cases in neighbouring regions. It remains to be determined whether the increasing incidence of human knowlesi malaria cases in southern Thailand [44] is linked to the northwards movement of macaques. Therefore, interventions and surveillance targeting NHP malaria must go beyond current efforts, and such ecological insights could support more effective surveillance and control strategies. Advancements in aerial imaging, geotracking devices and bioacoustics provide new opportunities for macaque monitoring, particularly in dense forests and remote areas [45–48]. For vector surveillance, a novel approach of canopy-level sampling could enhance the detection of uncommon vector species, offering insights into lesser-known transmission cycles in the forest canopy [49].

## Conclusions

These findings underscore the benefits of an integrative approach, such as the One Health framework, which fosters interdisciplinary collaboration across the human, animal and environmental health sectors. The relevant stakeholders from different sectors should collaborate and acknowledge the distinct threats highlighted by molecular technology by establishing robust monitoring, early warning and control strategies for both parasitology and virology disciplines at the wildlife and public-health levels. This approach is essential for understanding the full spectrum of disease transmission dynamics and developing comprehensive prevention strategies that address not only human health but also the health of wildlife and the environments that sustain them. Integrating insights from epidemiology, ecology, veterinary science and public health will be key to controlling zoonotic diseases such as *P. knowlesi* and reducing their impact on both human and animal populations. Although it is a common hypothesis that human *Plasmodium knowlesi* infections are more likely to occur in areas with infected macaque reservoirs, this study provides novel quantitative evidence of a spatiotemporal correlation between high *P. knowlesi* prevalence in macaques and increased occurrence of human *P. knowlesi* cases. These findings reinforce long-standing hypotheses that close proximity between macaque populations infected with *P. knowlesi* and human settlements increases the risk of spillover infections in humans, and zoonoses cannot be adequately addressed through isolated, single-discipline efforts. This study offered novel quantitative and spatiotemporal evidence that has been limited in previous research.

## Supplementary Information

Supplementary Material 1. Additional file: Fig S1. The number of inter-host sampling site pairs was calculated based on Euclidean distances between human, macaque, and mosquito sampling locations, up to a maximum distance of 40 km. Visualizations of site pairings within 10 km, 20 km, and 40 kmspatial proximity constraints. Black lines indicate the connections between paired sites. Additional file 1: Text S1. Formulas to calculate the historical human knowlesi malaria cases within specific spatial radius and time lead. Additional file 1: Fig S2. Schematics detailing how the number of historical human knowlesi malaria cases were counted based on radius and time lead. Additional file 1: Fig S3. Schematics detailing how the backward and forward cumulative number of historical human knowlesi malaria cases were counted based on radius and time lead. Additional file: Table S1. Median and interquartile range of the closest spatial proximitybetween each sampling site typeand the nearest site of another type. Distances represent the nearest-neighbour relationship between all sites of type X and any site of type Y. Additional file 1: Fig S4 Spatiotemporal correlation between the proportion of *P. knowlesi* positive macaques and the cumulative number of reported human *P. knowlesi* cases, evaluated across different spatial radii and time leads. Panel A shows the correlation involving backward cumulative sum of human knowlesi malaria case numbers from 0 down to 12-month leads. Panel B shows the correlation involving forward cumulative sum of human knowlesi malaria case numbers from 0 up to + 12-month leads. Negative month leads indicate lags, i.e. the number of months counted backward from the macaque sampling month. “NA” indicates that the correlation coefficient could not be computed due to the absence of reported human cases within the specified radius and time window. Panels C and D show statistical significance maps for the backwardand forward cumulative correlations. Additional file 1: Fig S5. Correlation plots of each sampling outcomes against the predicted maps

## Data Availability

The code and data generated in this study are available at https://github.com/WKPhang/pk-multi-pronged-peninsular-malaysia-lrgs. The historical human knowlesi malaria case data used in this study are not publicly available owing to confidentiality and ethical restrictions. Access to these data can be requested from the Disease Control Division, Ministry of Health, Malaysia, on reasonable request. Other data used to support the analysis in this study are available from the corresponding authors on reasonable request.

## Abbreviations

Adj.: Adjusted
ACD: Active case detection
*An*.: *Anopheles*
ARRIVE: Animal Research: Reporting of in Vivo Experiments
DNA: Deoxyribonucleic acid
DWNP: Department of Wildlife and National Parks
FDR: False discovery rate
HLC: Human landing catch
*ITS2*: Internal transcribed spacer 2
MBS: Mass blood survey
MREC: Medical Research and Ethics Committee
*n*: Frequency (sample count)
NHP: Non-human primate
NMRR: National Medical Research Register
*P.*: *Plasmodium*
PCD: Passive case detection
PCR: Polymerase chain reaction
rRNA: Ribosomal ribonucleic acid
*rs*: Spearman’s correlation coefficient
UM IACUC: Universiti Malaya Institutional Animal Care and Use Committee
WHO: World Health Organization
WDSP: Wildlife Disease Surveillance Programme
XGBOOST: Extreme Gradient Boosting
