# Supplementary material for: Multi-pronged surveillance to understand the spatiotemporal correlations among macaques, vectors and humans in Plasmodium knowlesi malaria transmission
Source: Parasit Vectors. 2025 Oct 29;18:439. doi: 10.1186/s13071-025-07082-6 (PMC12573820; doi:10.1186/s13071-025-07082-6)
Supplement: Supplementary file 1 — Supplementary Material 1. Additional file: Fig S1. The number of inter-host sampling site pairs was calculated based on Euclidean distances between human, macaque, and mosquito sampling locations, up to a maximum distance of 40 km. Visualizations of site pairings within 10 km, 20 km, and 40 kmspatial proximity constraints. Black lines indicate the connections between paired sites. Additional file 1: Text S1. Formulas to calculate the historical human knowlesi malaria cases within specific spatial radius and time lead. Additional file 1: Fig S2. Schematics detailing how the number of historical human knowlesi malaria cases were counted based on radius and time lead. Additional file 1: Fig S3. Schematics detailing how the backward and forward cumulative number of historical human knowlesi malaria cases were counted based on radius and time lead. Additional file: Table S1. Median and interquartile range of the closest spatial proximitybetween each sampling site typeand the nearest site of another type. Distances represent the nearest-neighbour relationship between all sites of type X and any site of type Y. Additional file 1: Fig S4 Spatiotemporal correlation between the proportion of P. knowlesi positive macaques and the cumulative number of reported human P. knowlesi cases, evaluated across different spatial radii and time leads. Panel A shows the correlation involving backward cumulative sum of human knowlesi malaria case numbers from 0 down to 12-month leads. Panel B shows the correlation involving forward cumulative sum of human knowlesi malaria case numbers from 0 up to + 12-month leads. Negative month leads indicate lags, i.e. the number of months counted backward from the macaque sampling month. “NA” indicates that the correlation coefficient could not be computed due to the absence of reported human cases within the specified radius and time window. Panels C and D show statistical significance maps for the backwardand forward cumulative correlations. Additiona [file 13071_2025_7082_MOESM1_ESM.pdf]

Additional File 1

Multi-pronged surveillance to understand the spatiotemporal correlations among macaques, vectors, and humans in *Plasmodium knowlesi* malaria transmission

Wei Kit Phang[1,2,3], Nantha Kumar Jeyaprakasam[4], Sandthya Pramasivan[1,3], Zailiza Binti Suli[5], Jenn Zhueng Tam[5], Mohd Hafizi Bin Abdul Hamid[5], Mohd Lutfi Bin Abdullah[6], Anis Adlina Binti Isman Rohimly[6], Norsharina Binti Ashrat[6], Ting-Wu Chuang[7]*, Wang Nguitragool[2,3], Indra Vythilingam[1], Yee Ling Lau[1]*

[1]Department of Parasitology, Faculty of Medicine, Universiti Malaya, Kuala Lumpur, Malaysia.

[2]Department of Molecular Tropical Medicine and Genetics, Faculty of Tropical Medicine, Mahidol University, Bangkok, Thailand.

[3]Mahidol Vivax Research Unit, Faculty of Tropical Medicine, Mahidol University, Bangkok, Thailand.

[4]Biomedical Science Program, Center for Toxicology and Health Risk Studies, Faculty of Health Sciences, Universiti Kebangsaan Malaysia, Kuala Lumpur, Malaysia.

[5]Disease Control Division, Ministry of Health Malaysia, Putrajaya, Malaysia.

[6]National Wildlife Forensic Laboratory, Ex-Situ Conservation Division, Department of Wildlife and National Parks Peninsular Malaysia, Kuala Lumpur, Malaysia.

[7]Department of Molecular Parasitology and Tropical Diseases, School of Medicine, College of Medicine, Taipei Medical University, Taipei, Taiwan.

*Correspondence: chtingwu@tmu.edu.tw; yeelinglau@um.edu.my

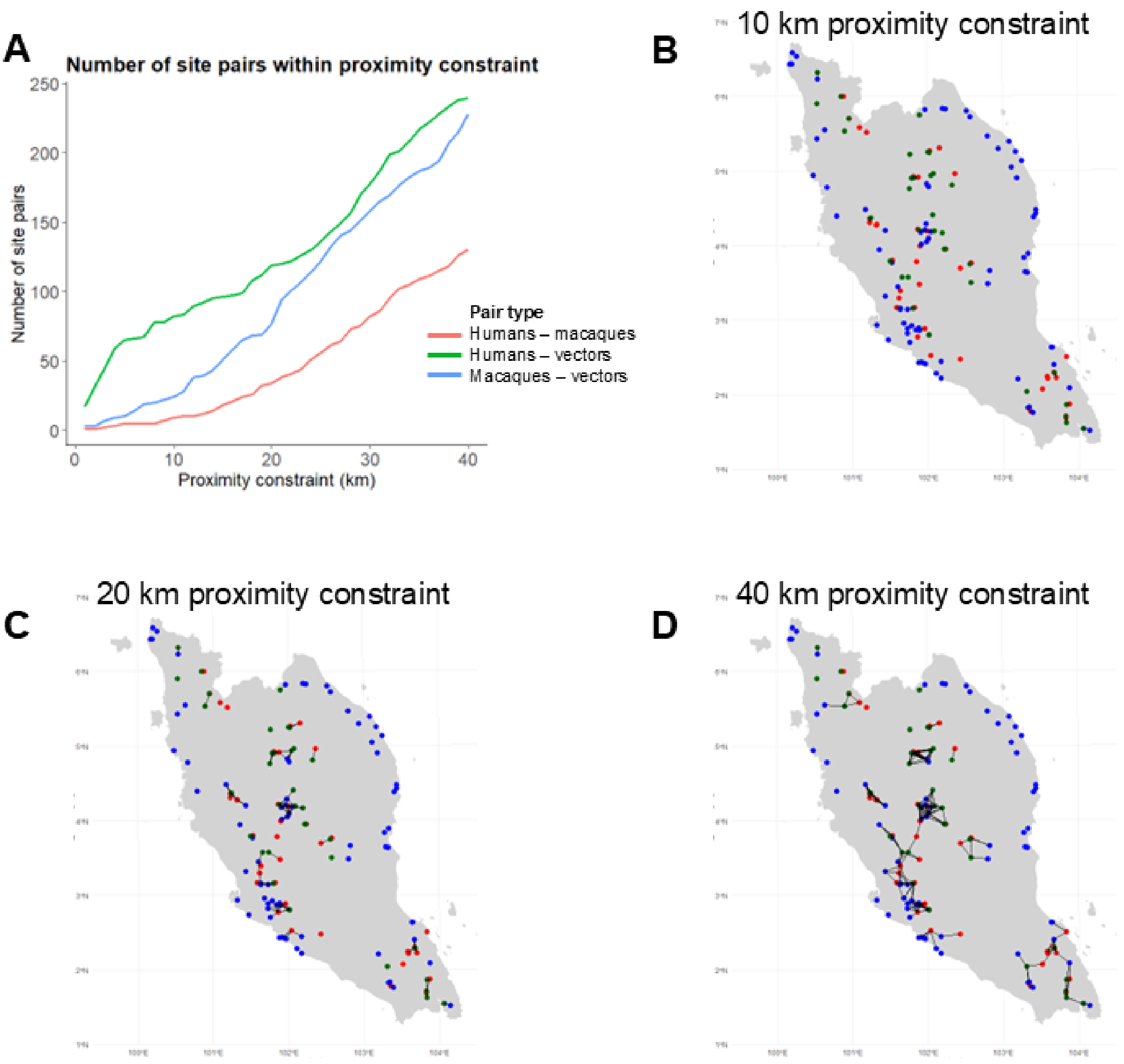

Additional file: Fig. S1. The number of inter-host sampling site pairs was calculated based on Euclidean distances between human, macaque, and mosquito sampling locations, up to a maximum distance of 40 km (A). Visualizations of site pairings within 10 km (B), 20 km (C), and 40 km (D) spatial proximity constraints. Black lines indicate the connections between paired sites.

Additional file 1: Text S1. Formulas to calculate the historical human knowlesi malaria cases within specific spatial radius and time lead.

1. Formula to calculate the number of historical human knowlesi malaria cases within specific spatial radius and time lead

$$C_{r,t}(s) = \sum \mathrm{I}(\mathrm{d}(\mathrm{s}, \mathrm{x_i}) \leq \mathrm{r} \wedge (t_i - t_0) = t)$$

Where,

r is radius from sampling site (e.g., 1 km, 2 km)

t is specific time lead (months)

s is spatial coordinates of the sampling site

x_i is spatial coordinates of of the *i*th human case

$d(s, x_i)$: Distance between sampling site s and case location x_i

t_0 is sampling month

I (…) is indicator function which returns 1 when the conditions, $d(s, x_i) \leq r$ and $(t_i - t_0) = t$, are satisfied, and 0 otherwise.

2. Formulas to calculate the backward and forward cumulative number of historical human knowlesi malaria cases within specific spatial radius and time lead

$$B(r, \Delta t) = \sum_{i = \Delta t}^{0} C(r, t_0 + i), \qquad \text{for } \Delta t \leq 0$$

$$F(r, \Delta t) = \sum_{i=0}^{\Delta t} C(r, t_0 + i), \qquad \text{for } \Delta t \geq 0$$

Where,

r is radius from sampling site (e.g., 1 km, 2 km)

t_0 is sampling month

Δt is time lead (e.g., -2, -1, 0, 1, 2 months)

C(r, t) is number of human *P. knowlesi* cases within radius *r* at time *t*

B(r, Δt) is backward cumulative number of cases

F(r, Δt) is forward cumulative number of cases

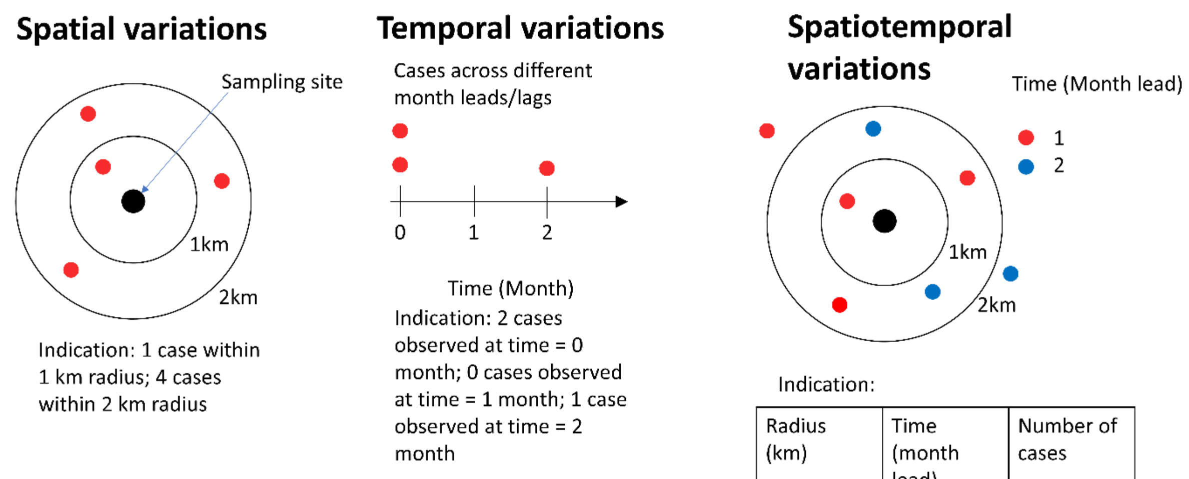

Radius (km)	Time (month lead)	Number of cases
1	1	1
1	2	0
2	1	3
2	2	2

Additional file 1: Fig. S2. Schematics detailing how the number of historical human knowlesi malaria cases were counted based on radius and time lead.

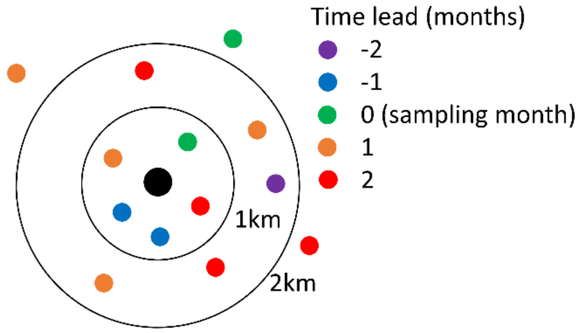

Backward cumulative number of cases indication

Radius (km)	Time lead (months)	Number of cases
1	0	1
1	-1	3
1	-2	3
2	0	1
2	-1	3
2	-2	4

Forward cumulative number of cases indication

Radius (km)	Time lead (months)	Number of cases
1	0	1
1	1	2
1	2	3
2	0	1
2	1	4
2	2	7

Additional file 1: Fig. S3. Schematics detailing how the backward and forward cumulative number of historical human knowlesi malaria cases were counted based on radius and time lead.

Additional file 1: Table S1. Median and interquartile range of the closest spatial proximity (in km) between each sampling site type (X) and the nearest site of another type (Y). Distances represent the nearest-neighbour relationship between all sites of type X and any site of type Y.

X	Y	Closest spatial proximity between each X sampling site and any Y site	
		Median (km)	Interquartile range (km)
Humans	Macaques	21.5	14.3 – 28.9
Humans	Vectors	3.0	0.7 – 19.9
Macaques	Humans	32.7	18.8 – 59.5
Macaques	Vectors	24.9	11.3 – 72.3
Vectors	Humans	4.17	1.29 – 14.3
Vectors	Macaques	19.4	11.1 – 28.3

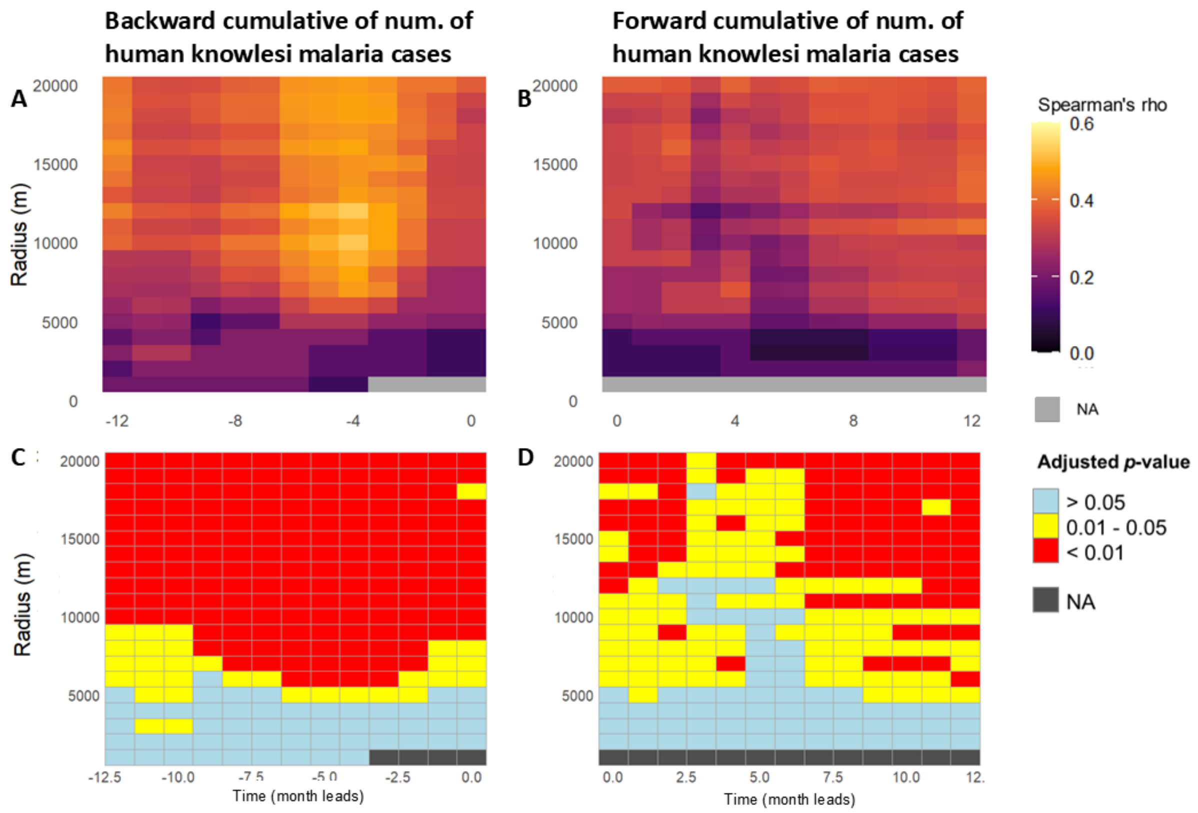

Additional file 1: Fig. S4 Spatiotemporal correlation between the proportion of *P. knowlesi* positive macaques and the cumulative number of reported human *P. knowlesi* cases, evaluated across different spatial radii and time leads. Panel A shows the correlation involving backward cumulative sum of human knowlesi malaria case numbers from 0 down to –12-month leads (A). Panel B shows the correlation involving forward cumulative sum of human knowlesi malaria case numbers from 0 up to +12-month leads (B). Negative month leads indicate lags, i.e., the number of months counted backward from the macaque sampling month. "NA" indicates that the correlation coefficient could not be computed due to the absence of reported human cases within the specified radius and time window. Panels C and D show statistical significance maps for the backward (C) and forward cumulative correlations (D).

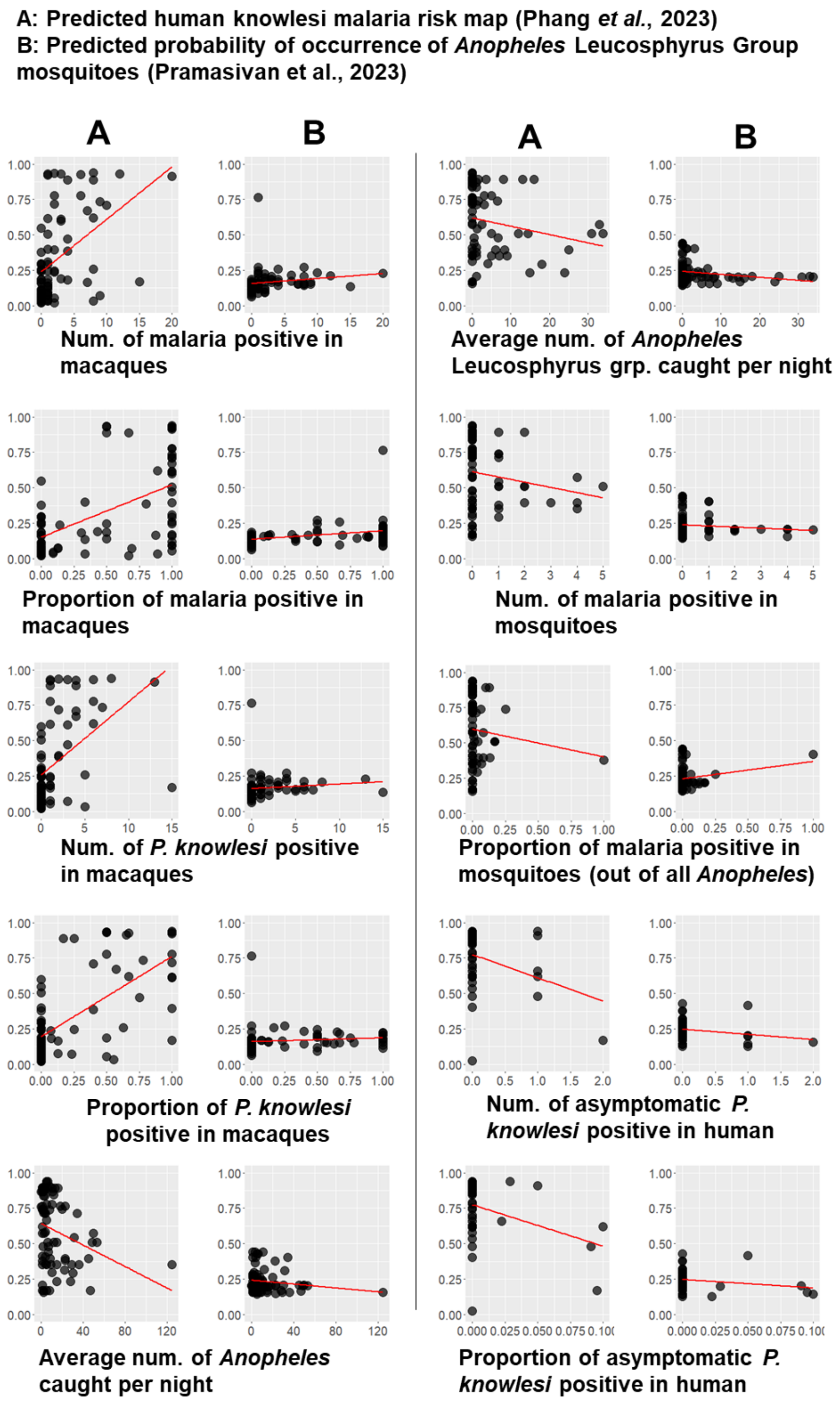

Additional file 1: Fig. S5. Correlation plots of each sampling outcomes against the predicted maps.
